# *De novo* assembly and characterization of the root transcriptome of *Aegilops variabilis* during an interaction with the cereal cyst nematode

**DOI:** 10.1186/1471-2164-13-133

**Published:** 2012-04-11

**Authors:** De-Lin Xu, Hai Long, Jun-Jun Liang, Jie Zhang, Xin Chen, Jing-Liang Li, Zhi-Fen Pan, Guang-Bing Deng, Mao-Qun Yu

**Affiliations:** 1 Chengdu Institute of Biology, Chinese Academy of Sciences, Chengdu, Sichuan, China; 2 Graduate University of the Chinese Academy of Sciences, Beijing, China

## Abstract

**Background:**

*Aegilops variabilis* No.1 is highly resistant to cereal cyst nematode (CCN). However, a lack of genomic information has restricted studies on CCN resistance genes in *Ae*. *variabilis* and has limited genetic applications in wheat breeding.

**Results:**

Using RNA-Seq technology, we generated a root transcriptome at a sequencing depth of 4.69 gigabases of *Ae. variabilis* No. 1 from a pooled RNA sample. The sample contained equal amounts of RNA extracted from CCN-infected and untreated control plants at three time-points. Using the Trinity method, nearly 52,081,238 high-quality trimmed reads were assembled into a non-redundant set of 118,064 unigenes with an average length of 500 bp and an N50 of 599 bp. The total assembly was 59.09 Mb of unique transcriptome sequences with average read-depth coverage of 33.25×. In BLAST searches of our database against public databases, 66.46% (78,467) of the unigenes were annotated with gene descriptions, conserved protein domains, or gene ontology terms. Functional categorization further revealed 7,408 individual unigenes and three pathways related to plant stress resistance.

**Conclusions:**

We conducted high-resolution transcriptome profiling related to root development and the response to CCN infection in *Ae*. *variabilis* No.1. This research facilitates further studies on gene discovery and on the molecular mechanisms related to CCN resistance.

## Background

Cereal cyst nematode (causal agent *Heterodeta avenae*) causes cereal disease in many regions of the world [1-5], and results in economic losses of billions of dollars annually [6]. Although CCNs have caused serious economic losses over the last 40 years [1], only a few CCN resistance genes have been genetically mapped on the genomes of wheat (*Cre1* and *Cre8*) and its relatives, such as those in the genus *Aegilops* (*Cre2*-7), *Secale cereale* (*CreR*) (reviewed in Smiley and Nicol (2009) [7]) and *Hordeum vulgare* (*Ha1-4*) (reviewed in Bakker et al. (2006) [8]). The molecular mechanism of CCN resistance remains unknown.

Members of the genus *Aegilops* readily hybridize with bread wheat as the male parent [9]. *Aegilops* species are valuable genetic resources for breeding for disease resistance in wheat; for example, for resistance to *Cochliobolus sativus* (spot blotch), *Tilletia indica* (*Karnal bunt*), and powdery mildew [10,11]. *Ae. variabilis* accession No.1 (2n = 4*x* = 28, UUS^v^S^v^) (syn. *Triticum peregrinum* (Hack In J. Fraser) Marie & Hackel) was reported to harbor resistance genes to both CCN and root knot nematode (*Meloidogyne naasi*) [12,13]. A greater understanding of the mechanism of CCN resistance in *Ae. variabilis* is necessary for wheat breeding. However, the major barrier against using genomic approaches to improve *Ae. variabilis* is that the genome sequence, cDNA libraries, EST databases, and microarray platform information are not available [14].

Recent developments in RNA-Seq technology have enabled very efficient probing of transcriptomic data [15-19]. This method not only detects transcripts that correspond to existing genomic sequences, but it can also be used for *de novo* assembly of short reads for gene discovery and expression profiling in organisms for which there is no reference genome [17,20-26].

In the present study, we analyzed the root transcriptome of *Ae. variabilis* using RNA-Seq technology. We used two methods, SOAPdenovo and Trinity, for *de novo* assembly of the transcriptome, and compared their results. Characterization of the transcriptome data assembled by Trinity give a high-resolution insight into the genes involved in several major metabolic pathways associated with root development and plant defense. This research will serve as a public information platform for further studies on the evolution and function of genes in *Ae. variabilis*, and provides a thorough insight into the gene expression profiles associated with the response to CCN infection in *Ae. variabilis*.

## Methods

### Plant material and pathogen infection

*Ae. variabilis* accession No.1 was used for transcriptomic profiling of genes expressed in roots. Grains of *Ae. variabilis* No.1 were surface-sterilized in a solution containing 3% (v/v) hypochlorite and 0.01% (v/v) Tween 20 for 5 min and rinsed three times with sterile water [27]. The seeds were germinated in Petri dishes (5-cm diameter) on wet paper at 20 C under a 16-h light/8-h dark photoperiod. After 10 days, seedlings were divided into two groups. One group was inoculated with 1,000 second-stage juveniles (J2) of CCN per plant, and the other group (negative control) was not inoculated with CCN [28,29]. Thirty hours after inoculation, the roots were thoroughly washed three times with sterile water (each 10 min) to remove CCNs adhering to roots. Then, plants were transplanted into 500-ml glass containers filled with sterilized perlite, and were grown at 20 C under a 16-h light/8-h dark photoperiod. These conditions prevented further CCN penetration and ensured synchronized development of syncytia [27,30].

### RNA isolation

Successful CCN inoculation was confirmed by observing roots under a microscope (Additional file 1). Roots of CCN-infected and non-infected plants were sampled at 30 hpi (hours post inoculation), 3 dpi (days post inoculation) and 9 dpi for RNA extraction [27,31,32]. Each sample consisted of 15 individuals. Total RNA was extracted with a Biomiga RNA kit according to the manufacturer’s protocol (Biomiga, San Diego, CA, USA). The concentration and quality of each RNA sample was determined using a NanoDrop 2000™ micro-volume spectrophotometer (Thermo Scientific, Waltham, MA, USA). Equal amounts of total RNA from each sample were pooled to construct the cDNA library. Pooling is a cost-effective strategy when the primary research goal is to identify gene expression profiles. This strategy was well-justified based on statistical and practical considerations [33-35].

### Construction of cDNA library and Illumina deep-sequencing

The cDNA library was constructed using an mRNA-Seq assay for paired-end transcriptome sequencing. The library construction and sequencing were performed by the Beijing Genomics Institute (BGI)-Shenzhen, Shenzhen, China. Briefly, mRNA was enriched from 20 μg total RNA using oligo dT magnetic beads, and was then cleaved into 200–700 nt fragments by incubation with RNA Fragmentation Reagent. The fragmented mRNA was converted into double-stranded cDNA by priming with random hexamer-primers, purified with a QiaQuick PCR extraction kit (QIAGEN Inc., Valencia, CA, USA), and then washed with EB buffer for end repairing and single nucleotide adenine addition. Finally, sequencing adaptors were ligated onto the fragments, and the required fragments were purified by agarose gel electrophoresis and enriched by PCR amplification to construct the cDNA library. The library was loaded onto the channels of an Illumina HiSeq™ 2000 instrument for 4 gigabase in-depth sequencing, which was used to obtain more detailed information about gene expression. Each paired-end library had an insert size of 200–700 bp. The average read length of 90 bp was generated as raw data. The data sets are available at the NCBI SRA database with the accession number of SRA050454.

### *De novo* assembly and sequence clustering

The clean reads were obtained from raw data by filtering out adaptor-only reads, reads containing more than 5% unknown nucleotides, and low-quality reads (reads containing more than 50% bases with Q-value ≤ 20). Then *de novo* assembly of the clean reads was performed to generate non-redundant unigenes. We used two methods for *de novo* assembly; SOAPdenovo 63mer-V1.05 [36] with optimized *k*-mer length of 41, and the Trinity method [19] with optimized *k*-mer length of 25.

Sequence directions of the resulting unigenes were determined by performing BLASTX searches against protein databases, with the priority order of NR (non-redundant protein sequences in NCBI), Swiss-Prot, Kyoto Encyclopedia of Genes and Genomes database (KEGG), and COG (E-value ≤ 1e-5) if conflicting results were obtained. ESTScan software [37] was also used to determine the directions of sequences that were not aligned to those in any of the databases mentioned above.

The expression levels of unigenes were measured as the number of clean reads mapped to its sequence. The number of clean reads mapped to each annotated unigene was calculated and then normalized to RPKM (reads per Kb per million reads) with ERANGE3.1 software [18] and adjusted by a normalized factor [38].

### Functional categorization of unigenes

The unigenes assembled by the Trinity method that were longer than 200 bp were annotated according to their sequence similarity to previously annotated genes. We used sequence-based and domain alignments to compare sequences. Sequence-based alignments were performed against three public databases (NR, Swiss-Prot, and KEGG; significant thresholds of E-value ≤ 1e-5). Domain-based alignments were carried out against the COG database at NCBI with a cut-off E-value of ≤1e-5.

The resulting BLAST hits were processed by Blast2GO software [39] to retrieve associated Gene Ontology (GO) terms describing biological processes, molecular functions, and cellular components [40]. By using specific gene identifiers and accession numbers, Blast2GO produces GO annotations as well as corresponding enzyme commission numbers (EC) for sequences with an E-value ≤1e-5.

KEGG mapping was used to determine the metabolic pathways [41,42]. The sequences with corresponding ECs obtained from Blast2GO were mapped to the KEGG metabolic pathway database. To further enrich the pathway annotation and to identify the BRITE functional hierarchies, sequences were also submitted to the KEGG Automatic Annotation Server (KAAS) [43], and the single-directional best hit information method was selected. KAAS annotates every submitted sequence with KEGG orthology (KO) identifiers, which represents an orthologous group of genes directly linked to an object in the KEGG pathways and BRITE functional hierarchy [43,44]. Therefore, these methods incorporate different types of relationships that exist in biological systems (i.e. genetic and environmental information processing, cellular processes, and organism systems).

## Results

### Transcriptome sequencing, *de novo* assembly, and sequence analysis

We constructed a cDNA library of pooled RNA samples to generate a transcriptomic view of genes expressed in the root of uninfected and CCN-infected *Ae. variabilis*. Approximately 4,687,311,420 base pairs of raw data were generated, yielding a total of 54,267,786 clean reads that were 90 bp in length (Table 1). Of the clean reads, 91.63% had a Phred quality score of ≤ Q20 level (error probability of 0.01).

**Table 1:** Summary of de novo sequence assembly

	**Sequences (n)**	**Base pairs (Mbp)**	**Length range (bp)**	**Mean length (bp)**	**N50 (bp)**
Clean reads	52,081,238	4,687.31	90-90	90	90
SOAP contigs	336,641	60.21	60-3911	200	229
Trinity contigs	481,672	92.50	75-3696	192	250
SOAP unigenes	130,487	45.86	150-4113	351	392
Trinity unigenes	118,064	59.09	200-4214	500	599

All trimmed reads were *de novo* assembled by SOAPdenovo and Trinity programs (Table 1). SOAPdenovo produced 336,641 contigs of 60 to 3,911 bp with an average length of 200 bp and an N50 of 229 bp (i.e., 50% of the assembled bases were incorporated into contigs of 229 bp or longer). The majority of the contigs were shorter than 200 bp (71.97%), and 2,722 contigs (0.81%) were longer than 1,000 bp. Trinity generated 481,672 contigs ranging from 75 to 3696 bp with an average length of 192 bp and an N50 of 250 bp. Similar to the SOAPdenovo assembly, most contigs were shorter than 200 bp (79.65%) but there was a greater number of longer contigs—11,394 contigs (2.37%) were longer than 1000 bp. The size distribution of these contigs is shown in Table 2. A total of 130,487 unigenes were further generated by SOAPdenovo. The unigenes had an average length of 351 bp and an N50 of 392 bp. Among the unigenes, 37,828 (28.99%) were shorter than 200 bp and 4,702 (3.60%) were longer than 1,000 bp. The Trinity method generated fewer unigenes (118,064). These unigenes had an average length of 500 bp and an N50 of 599 bp. Among the unigenes, 64,330 unigenes (54.49%) were 200 to 400 bp in length. There were no unigenes shorter than 200 bp and 9.00% (10,622) of all generated unigenes were longer than 1,000 bp (Table 2).

**Table 2:** Length and number distribution of the unigenes and contigs

**Length range (bp)**	**SOAPdenovo**		**Trinity**	
	**Contig No.**	**Unigene No.**	**Contig No.**	**Unigene No.**
<200	242,275	37,828	383,651	0
200-299	51,635	42,043	41,094	41,031
300-399	19,366	19,062	18,886	23,299
400-499	9,163	10,457	9,454	13,917
500-999	11,480	16,395	17,193	29,195
1000-1999	2,497	4,120	8,913	10,027
2000-3000	208	505	1969	572
>3000	17	77	512	23
Total	336,641	130,487	481,672	118,064

**Figure 1:**
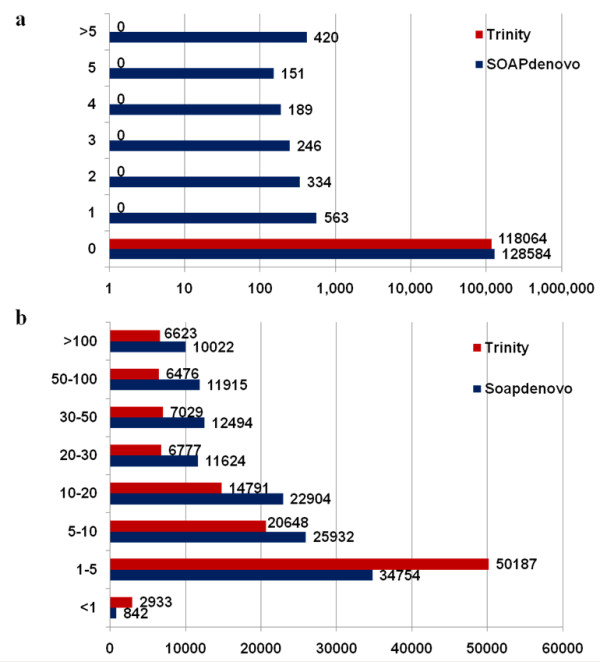
**Quality of the unigenes assembled.a**, Length ratio of the gap to unigenes assembled by SOAPdenovo and Trinity method, respectively. The x-axis indicates the number of unigenes containing gaps; the y-axis indicates the percentage of the gap length to unigenes length. **b**, A histogram of the average read-depth coverage for unigenes. The x-axis indicates the number of unigenes, and the y-axis indicates folds distribution of read-depth coverage.

To assess the quality of the data set, we evaluated the assembled unigenes to determine the presence and length of gaps in the sequences. The analysis showed that 1.56% of the unigenes assembled by SOAPdenovo contained gaps, whereas those assembled by Trinity contained no gaps (Figure 1a).

Because there is no transcriptome profile of *Ae*. *variabilis* available for comparison, we used a web-based tool, ESTcal [45], to evaluate the depth and breadth of our data set. The read-depth coverage for 35.29% of SOAPdenovo-generated unigenes and for 22.79% of Trinity-generated unigenes was greater than 20 fold (Figure 1b), with an average read-depth coverage of 33.54-fold and 33.25-fold, respectively.

### Annotation and classification of the root transcriptome in *ae. Variabilis*

**Figure 2:**
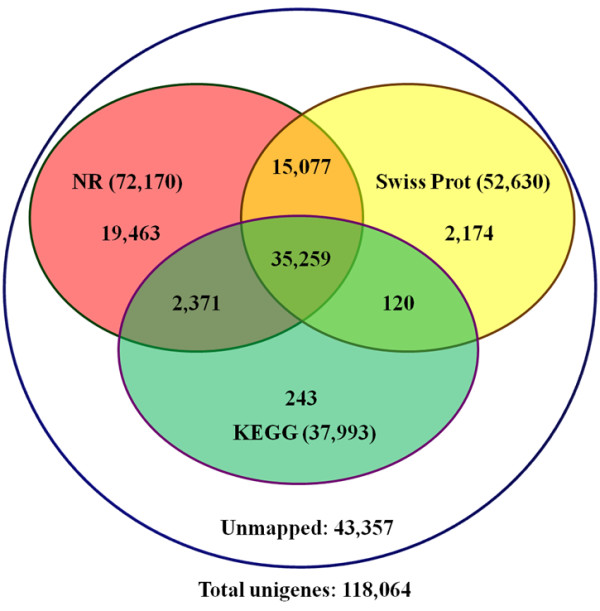
**Detection of Homologous genes in public databases.** The numbers of annotated and unmapped unigenes were indicated in the ellipses, respectively.

To validate and annotate the assembled unigenes, the 118,064 unigenes generated by Trinity were subjected to BLASTX searches (E-value ≤ 1e-5) against public protein databases. As a result, 72,170 (61.13%), 52,630 (44.58%), and 37,993 (32.18%) unigenes had homologous sequences in NR, Swiss-Prot, and KEGG databases, respectively (Figure 2). Among the unigenes, 50,336 (42.63%) were synchronously annotated by NR and Swiss-Prot, 37,630 (31.87%) by NR and KEGG, and 35,379 (29.97%) by Swiss-Prot and KEGG, and 35,259 (29.86%) unigenes were simultaneously annotated by all three databases. Also, 43,357 (36.72%) unigenes showed no homology to known sequences deposited in these databases (Figure 2).

### GO classifications

**Figure 3:**
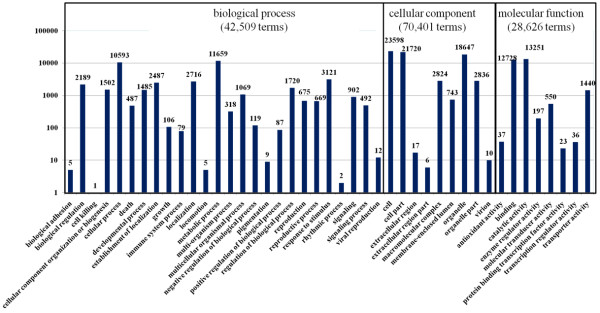
**GO annotations of unigenes by Blast2GO.** The x-axis indicated the sub-categories and the y-axis indicates the number of unigenes, and the unigene numbers assigned with same GO terms were indicated on the top of the rectangle bars.

The unigenes homologous to known sequences in NR, Swiss Prot, and KEGG were further annotated with GO terms using Blast2GO [39]. A total of 31,789 (26.93%) unigenes were assigned 141,172 GO term annotations, which could be classified into three categories; biological process, molecular function, and cellular component. The biological process category consisted of 42,509 GO terms, which were assigned to 17,953 (15.21%) unigenes. The molecular function category consisted of 70,401 GO terms, which were assigned to 23,381 (19.80%) unigenes, and the cellular component category consisted of 28,262 GO terms, which were assigned to 23,798 (20.16%) unigenes. In addition, 12,340 (10.45%) unigenes were simultaneously annotated in all three categories (Figure 3). Within the biological process category, unigenes were assigned to “metabolic process” (11,659 terms), “cellular process” (10,593 terms), “response to stimulus” (3,121 terms), “localization” (2,716 terms), and “establishment of localization” (2,487 terms). In the cellular component category, most unigenes were assigned to “cell” (23,598 terms), “cell part” (21,720 terms), and “organelle” (18,647 terms). In the molecular function category, the major GO terms were “catalytic activity” (13,251 terms) and “binding” (12,728 terms).

The five subcategories, “response to stimulus”, “death”, “immune system process”, “cell killing” and “antioxidant activity”, are all involved in resistance-related biological processes in the responses to abiotic and biotic stimulus/stress, based on their function explanations (Additional file 2).

### KEGG pathway mapping

To identify biological pathways activated in the root of *Ae. variabilis*, the assembled unigenes were annotated with Enzyme Commission (EC) numbers from BLASTX alignments against the KEGG database (E-value ≤ 1e-5). The assigned EC numbers were subsequently mapped to the reference canonical pathways. As a result, 37,993 unigenes (32.18% of 118,064) matched 57,975 members involved in 119 KEGG pathways (Additional file 3). Of the 37,993 unigenes, 9,596 were related to metabolic pathways, 4,815 to biosynthesis of secondary metabolites, 3,355 to spliceosome, 3,216 to plant-pathogen interaction, and 2,275 to ribosome.

Furthermore, 3,798 (including 3,712 individual unigenes) of the 57,975 members were sorted into the plant immune response pathways category, which includes plant-pathogen interaction, phosphatidylinositol signaling system, and ABC transporters (Additional file 3). These pathways are closely related to plant defense against biotic/abiotic stress.

### COG classification

**Figure 4:**
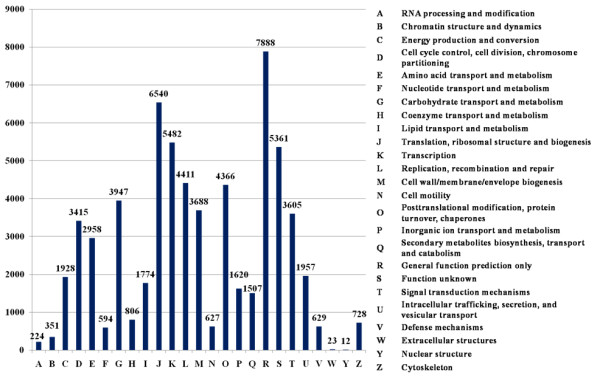
**COG function classification.** All unigenes were aligned to COGs database at NCBI to predict and category possible functions. Total of 28,126 unigenes were assigned to 25 classifications. The capital letters in x-axis indicates the COG categories as listed on the right of the histogram and the y-axis indicates the number of unigenes.

All assembled unigenes were further annotated based on COG category [46]. A total of 28,126 unigenes were assigned 64,441 functional annotations, which could be grouped into 25 functional categories (Figure 4). The largest category was “General function prediction only” (7,888 COG annotations, 12.24% of 64,441). Approximately 36.5% of the COG categories were associated with root development, including “Translation, ribosomal structure and biogenesis” (6,540, 10.15%), “Transcription” (5,482, 8.51%), “Posttranslational modification, protein turnover, chaperones” (4,366, 6.78%), “Cell wall/membrane/envelope biogenesis” (3,688, 5.72%) and “Cell cycle control, cell division, chromosome partitioning” (3,415, 5.30%), etc. In addition, 5,361 (8.32%) unigenes belonged to the “Function unknown” category.

The category of “Defense mechanisms” (629, 0.98%) is closely related to plant defense. The most abundant type of sequence in this category was ABC-type multidrug transport system [47]. A total of 486 unigenes belonged to ATPase and permease (Additional file).

In summary, 78,467 (66.46% of 118,064) unigenes were annotated in the four public databases. Among them, 74,707 unique unigenes were annotated in NR, Swiss-Prot, and KEGG databases through sequence-based alignments (Figure 2). Further, 22,522 unigenes were annotated in the COG database *via* domain-based alignments (Figure 3), which provided a further 3,760 annotated unigenes. Approximately one-quarter (18,762) of the annotated unigenes were simultaneously annotated with defined functional annotations in the four public databases. Their functional assignments are summarized in Additional file 5.

Among the annotated unigenes, 839 (0.17% of 118,064) showed high homology to sequences of nematode species, e.g. *Caenorhabditis elegans*, *Brugia malayi,* and *Globodera rostochiensis*, etc. (Additional file 6).

### Expression level

Gene expression levels were estimated by RPKM values. The distribution of RPKM values indicated that most genes were expressed at low levels. Among 118,064 unigenes, 31,484 (26.67%) had RPKM values of less than 1, and 96,687 (81.89%) had RPKM values of less than 10. The RPKM values of 2152 unigenes (1.82%) were greater than 100 (Additional file 5).

## Discussion

*Ae. variabilis* accession No.1 is a valuable resource for development of CCN-resistance in wheat breeding [12,13]. However, it is difficult to screen for genes associated with CCN resistance when genomic information is not available. Transcriptomic profiling provides abundant information for a wide range of biological studies. Transcriptomic data gives fundamental insights into biological processes. It can reveal gene expression profiles after experimental treatments or infection, and analyses of conserved orthologous genes can be used for phylogenomic purposes, etc. [48]. Here, we used high-throughput deep sequencing technology to profile the root transcriptome of *Ae. variabilis* using the Illumina HiSeq™ 2000 platform. To the best of our knowledge, this is the first report on this subject for *Ae. variabilis*. The cDNA library was constructed using pooled RNA samples from CCN-infected and non-infected plants at three time points. This maximized the number of expressed transcripts included in the analysis, especially those related to CCN resistance.

Accurate sequencing and reliable read assembly are essential for downstream applications of transcriptome data [49]. In this study, we used two popular assemblers, SOAPdenovo and Trinity, for *de novo* assembly of the transcriptomic data of *Ae. variabilis*. The SOAPdenovo program has been widely used in many studies [25,50], while the Trinity method is a newly developed tool. Trinity was reported to recover more full-length transcripts across a broad range of expression levels, and to provide a unified, sensitive solution for transcriptome reconstruction in species without a reference genome, similar to methods that rely on genome alignments [19]. The two methods showed similar average read-depth coverage values. SOAPdenovo produced more unigenes than Trinity; however, many of the sequences assembled by SOAPdenovo were shorter than 200 bp (37,828 out of 130,487). On the other hand, Trinity generated 118,064 unigenes, the unigenes did not contain gaps, and the average unigene length was nearly twice that of those produced by SOAPdenovo (mean length of 599 bp using Trinity, 351 bp using SOAPdenovo). Therefore, Trinity was a better approach than SOAPdenovo for assembly in this research.

The Roche 454 GS FLX platform produces long reads (≫400 bp), whereas the Illumina sequencer generates more reads with a shorter length (90 bp). In this study, however, most of the assembled unigenes (130,487 from SOAPdenovo (≥150 bp) or 118,064 from Trinity (≥200 bp)) achieved a higher coverage of ~33×. This indicates that short-read sequencing combined with an in-depth sequencing strategy and an effective assembly tool is an appropriate strategy to analyze transcriptome profiles.

Compared with other transcriptome studies, the length distribution of the 130,487 and 118,064 unigenes generated in this work tended towards shorter-length reads. There are several possible explanations for this. First, *Ae. variabilis* (2n = 4x = 28, UUS^v^S^v^) is an allotetraploid species of the tribe *Titiceae* and it has an enormously expanded repeated genome. This may present a substantial barrier to assembling short unigenes into long ones using current and upcoming sequencing technology [51,52]. Second, the total RNA for sequencing in our work was pooled from six samples, which may negatively affect read assembly [53]. The high dynamic range of mRNA expression is a problem for comprehensive *de novo* mRNA sequencing and assembly [50]. Third, high frequencies of alternative splicing and fusion events may have restricted the assembly of short sequences into longer ones [54,55]. Another important reason is that more than 80% of unigenes in this study were expressed at low levels. Therefore, there would be fewer reads corresponding to these unigenes for sequencing and for use in sequence assembly. Even so, the *de novo* transcriptome of *Ae. variabilis* provided abundant unigene information without gaps in sequences. This genetic data enriches the genomic resources for the tribe *Titiceae*.

A total of 7,408 individual unigenes (6.27% of 118,064) were associated with plant defense and resistance (Additional file 7). These unigenes could be classified into five GO sub-categories, three pathways, and a COG function group. More attention should be paid to the three pathways related to plant defense, which included 3712 unigenes. In the “plant-pathogen interaction” pathway, unigenes were mainly involved with the hypersensitive response, cell wall reinforcement, stomatal closure, and defense-related gene induction (Additional file 8). In the “phosphatidylinositol signaling system” pathway, unigenes were mainly related to reactions involving phosphatidylinositol and its derivatives (Additional file 9). In the “ABC transporters” pathway, unigenes were related to eukaryotic-type transporters only, such as the ABCA subfamily, ABCB subfamily, ABCC subfamily, ABCG subfamily, and other putative ABC transporters (Additional file 10). These pathways provide a starting point to explore the genes related to CCN resistance and to understand its molecular mechanism.

Interestingly, 839 unigenes showed high homology to genes from nematode species (Additional file 6), probably because the root had been invaded by CCNs. As there is no genomic information available for CCN, we cannot thoroughly filter sequences of *H. avenae* genes from the transcriptome database. However, the detection of CCN unigenes confirmed that the method used for CCN inoculation was successful. More importantly, these unigenes represent those expressed during the interaction with a resistant host. Therefore, this experimental system and the unigene dataset obtained from it build a platform for combining genetic, genomic, and expression information on the interaction between CCN and its host in future studies [56].

## Conclusions

This is the first report of transcriptome profiling of *Ae. variabilis* using high-throughput deep sequencing technology. The sequencing was at a depth of 4.69 gigabase pairs. A total of 118,064 unigenes were assembled and 78,467 unigenes were functionally annotated. By including RNA samples from CCN-infected plants, the dataset shown here may reveal important information about gene expression related to the plant response to, and defense against, CCN invasion. Consequently, the large number of transcriptomic sequences and their functional annotations will provide sufficient information to discover novel genes and to explore the molecular mechanism of CCN resistance in *Ae. variabilis.* Therefore, the results of this study will be useful for improving CCN resistance in wheat breeding programs.

## Competing interests

The authors declare that they have no competing interests.

## Authors’ contributions

MQY and HL conceived this study. DLX and HL designed the experimental plan, drafted and revised the manuscript. DLX, HL, JJL, JZ, XC, JLL, ZFP and GBD participated in sample collection, RNA preparation, performed experiments, analyzed and interpreted the sequence data. All authors read and approved the final manuscript.

## Supplementary Material

Additional file 1 **Roots invaded by CCN.** A prep-experiment confirmed the CCN J2 could parasitize plant root effectively before RNA extraction. Few hours later after the CCN inoculation, one nematode was detected being piercing root epidermis (Figure S1a). Utilizing the microscope, one CCN J2 was found invading into a root tip of plant, already (Figure S1b; part of the CCN cover was removed). Click here for file

Additional file 2 Resistance related unigenes from GO classification CCN. Click here for file

Additional file 3 KEGG pathway mapping. Click here for file

Additional file 4 Defending unigenes from COG alignment. Click here for file

Additional file 5 Unigene annotations in public databases. Click here for file

Additional file 6 Nematode-like unigenes list in the transcriptome database. Click here for file

Additional file 7 Resistance candidate unigenes in this study. Click here for file

Additional file 8 Unigenes involved in plant-pathogen interaction pathway. Click here for file

Additional file 9 Unigenes involved in phosphatidy linositol signaling system pathway. Click here for file

Additional file 10 Unigenes involved in ABC transporters pathway. Click here for file
