# Supplementary material for: De novo assembly and characterization of the root transcriptome of Aegilops variabilis during an interaction with the cereal cyst nematode
Source: BMC Genomics. 2012 Apr 11;13:133. doi: 10.1186/1471-2164-13-133 (PMC3439707; doi:10.1186/1471-2164-13-133)
Supplement: Additional file 8 — Unigenes involved in plant-pathogen interaction pathway. [file 1471-2164-13-133-S8.doc]

**Figure S1**


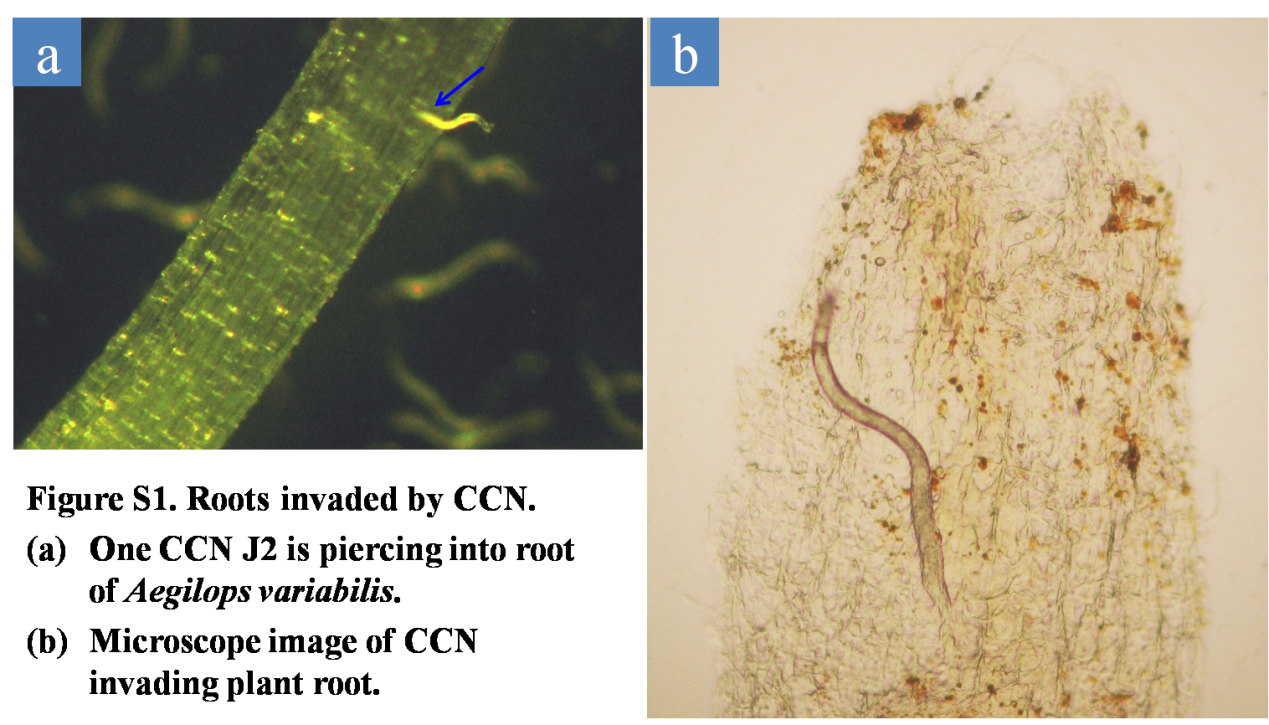


**Figure S1 Roots invaded by CCN.** A prep-experiment confirmed the CCN J2 could parasitize plant root effectively before RNA extraction. Few hours after the CCN inoculation, one nematode was detected being piercing root epidermis (Figure 1a). Utilizing the microscope, one CCN J2 was found invading into a root tip of plant, already (Figure 1b; part of the CCN cover was removed).
